# Characterization and Trypanocidal Activity of a Novel Pyranaphthoquinone

**DOI:** 10.3390/molecules22101631

**Published:** 2017-09-30

**Authors:** Elen Diana Dantas, Fabia Julliana Jorge de Souza, William Nascimento Litaiff Nogueira, Cláudia Cândida Silva, Pedro Henrique Antunes de Azevedo, Cícero Flávio Soares Aragão, Patricia Danielle Oliveira de Almeida, Mariana Filomena do Carmo Cardoso, Fernando de Carvalho da Silva, Eduardo Pereira de Azevedo, Euzébio Guimarães Barbosa, Emerson Silva Lima, Vitor Francisco Ferreira, Ádley Antonini Neves de Lima

**Affiliations:** 1 Pharmacy Department, Health Sciences Center, Universidade Federal do Rio Grande do Norte (UFRN), Natal RN 59012-570, Brazil; elendiana88@gmail.com (E.D.D.); fabiajulliana@gmail.com (F.J.J.d.S.); will_litaiff@hotmail.com (W.N.L.N.); pedrohenriqueantunesdeazevedo@hotmail.com (P.H.A.d.A.); cicero.aragao@yahoo.com.br (C.F.S.A.); euzebiogb@gmail.com (E.G.B.); 2 Crowfoot Group of X-ray Techniques, Universidade Estadual do Amazonas (UEA), Manaus AM 69065-020, Brazil; claudiacsbr@gmail.com; 3 Biological Activities Laboratory, Universidade Federal do Amazonas (UFAM), Pharmaceutical Sciences, Manaus AM 69080-900, Brazil; patt_danielle@hotmail.com (P.D.O.d.A.); eslima@ufam.edu.br (E.S.L.); 4 Laboratory of Synthesis of Bioactive Molecules, Organic Chemistry Department, Universidade Federal Fluminense (UFF), Niterói RJ 24020-141, Brazil; marianafcc83@hotmail.com (M.F.d.C.C.); gqofernando@vm.uff.br (F.d.C.d.S.); cegvito@vm.uff.br (V.F.F.); 5 Graduate Program in Biotechnology, Laureate International Universities—Universidade Potiguar (UnP), Natal RN 59056-000, Brazil; azevedoep@hotmail.com

**Keywords:** physicochemical characterization, IVS320, quinone, *T. cruzi*, Chagas disease, pyranaphthoquinone

## Abstract

Chagas disease is an endemic parasitic infection that occurs in 21 Latin American countries. New therapies for this disease are urgently needed, as the only two drugs available (nifurtimox and benznidazol) have high toxicity and variable efficacy in the disease’s chronic phase. Recently, a new chemical entity (NCE) named Pyranaphthoquinone (IVS320) was synthesized from lawsone. We report herein, a detailed study of the physicochemical properties and in vitro trypanocidal activity of IVS320. A series of assays were performed for characterization, where thermal, diffractometric, and morphological analysis were performed. In addition, the solubility, permeability, and hygroscopicity of IVS320 were determined. The results show that its poor solubility and low permeability may be due to its high degree of crystallinity (99.19%), which might require the use of proper techniques to increase the IVS320’s aqueous solubility and permeability. The trypanocidal activity study demonstrated that IVS320 is more potent than the reference drug benznidazole, with IC50/24 h of 1.49 ± 0.1 μM, which indicates that IVS320 has potential as a new drug candidate for the treatment of Chagas disease.

## 1. Introduction

Chagas disease, a parasite infection caused by the protozoan *Trypanosoma cruzi* (*T. cruzi*), is considered a health problem in Latin America due to inappropriate therapy and lack of an effective vaccine [1]. It is estimated that this infection affects about 8–9 million people in Latin America, causing about 10,000 deaths each year [2]. In the last decade, however, the migration of infected individuals with *T. cruzi* from endemic countries to non-endemic countries in North America, Europe, Asia, and Oceania has caused the spread of this disease around the world [3].

In the 1970s, nifurtimox and benznidazole appeared as the first effective drugs for the treatment of Chagas’ disease. The use of these drugs for long periods has been associated with several adverse effects [4]. In fact, nifurtinox has been discontinued due to serious side effects. Currently, benzinidazole (*N*-benzyl-2-nitro-1-imidazole acetamide) is the only drug available for the treatment of Chagas’ disease [5]. However, its low efficacy in the chronic phase of the disease, the high discontinuity of treatment due to its many side effects, and its low solubility have limited its clinical use [6].

Quinones represent an important class of biologically active molecules [7]. Naphthoquinones are a type of quinone that have a chemical structure based on the naphthalene ring [8]. The naphthoquinones (found in bacteria and fungi) have been extensively studied in recent years, not only because of their role in vital biochemical processes, but also due to their wide range of pharmacological properties such as antineoplastic [9,10,11], antimicrobial [11,12], insecticidal [13], antimalarial [14], anti-inflammatory [15] leishmanicidal [16,17], trypanocide [16], and antifungal [18].

**Figure 1:**
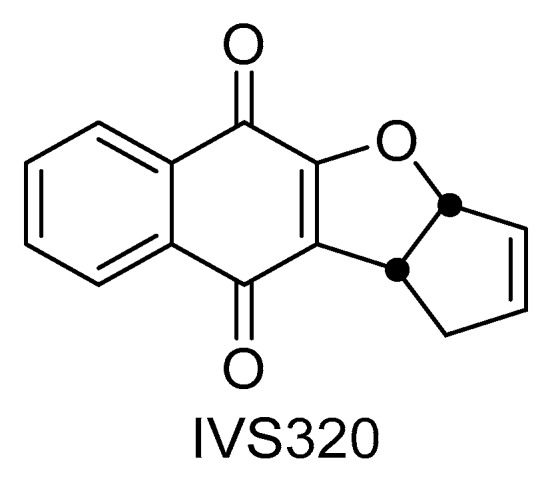
Chemical structure of IVS320 (3*a*,10*b*-dihydro-1*H*-cyclopenta[*b*]naphtho[2,3-*d*]furan-5,10-dione).

Recently, a pyranaphthoquinone (IVS320—Figure 1) was synthesized [19] and it has shown activity against *Candida albicans*, dermatophytes and *Cryptococcus* spp. [20]. However, IVS320’s trypanocidal activity is still unknown.

In this study, physicochemical characterization of IVS320 through X-ray Diffraction (XRD), Fourier-Transform Infrared spectroscopy (FTIR), X-ray Fluorescence by Dispersion of Energy (XRFDE), and thermal analysis such as Differential Scanning Calorimetry (DSC), Thermogravimetry (TG), and Differential Thermal Analysis (DTA) were performed. In addition, IVS320’s hygroscopicity, solubility, and partition coefficient were determined. Finally, the trypanocidal activity of IVS320 against tissue culture trypomastigotes forms of *T. cruzi* was investigated.

## 2. Results and Discussion

### 2.1. Thermal Analysis

**Figure 2:**
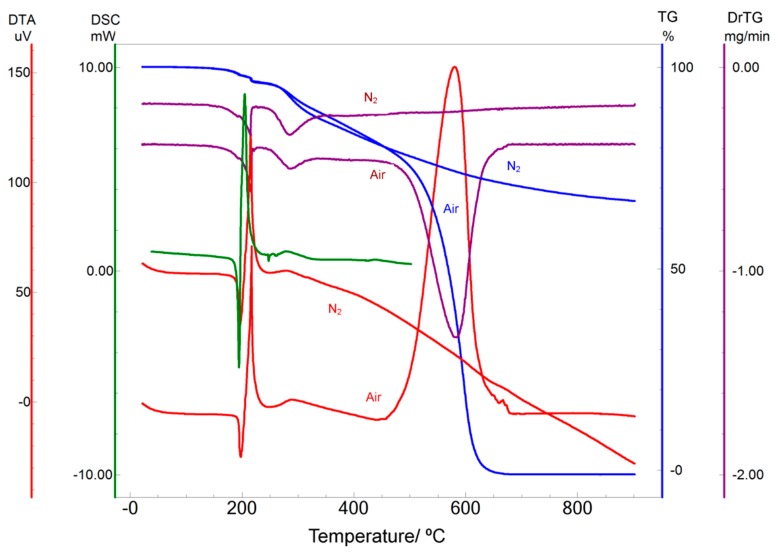
Differential scanning calorimetry (DSC), thermogravimetry TG/DrTG and differential thermal analysis (DTA) curves for IVS320 obtained at a heating rate of 10 °C·min^−1^ in a dynamic air and nitrogen atmospheres (50 mL·min^−1^).

The DSC curve for IVS320 (Figure 2) confirmed the three events characteristic of the thermal behavior of this material, where a sharp endothermic event is observed between 191 and 196 °C indicating IVS320 melting (*T*_peak_ 194 °C, Δ*H* −65.33 J·g^−1^). A second event is observed as an exothermic between 199 and 211 °C (*T*_peak_ 205 °C), followed by another exothermic event between 256 and 264 °C (*T*_peak_ 277 °C), which seems to be attributed to the recrystallization of IVS320, indicating a possible change to another crystalline form. 

By definition, polymorph is a compound that has at least two crystalline arrangements and although they have the same chemical composition, polymorphs usually exhibit different physicochemical properties. Therefore, aspects such as solubility, toxicity, and stability tend to change between the different crystalline forms [21]. Considering this information, it would be worthwhile investigating the different crystal forms of IVS320 by thermomicroscopy in a subsequent study.

When the thermal events of IVS320 are compared with other 2-Hydroxy-1,4-naphthoquinone (β-lapachone) derivatives with chemical structures similar to IVS320, some differences in the thermal events are observed. For instance, the thermal analysis of β-lapachone does not show any recrystallization event. Instead, two events are observed, where the first one is due to β-lapachone’s melting (T_peak_ 156, T_onset_ 156 and Δ*H* 122 J·g^−1^) and the second corresponds to its decomposition (256.3 °C and ∆H 69.4 J·g^−1^) [22].

The TG/DTG curves (Figure 2) show that IVS320 is thermally stable up to 150 °C, where three stages of mass loss can be observed. The first stage of mass loss (−4% decrease) occurred in the temperature range of 150–262 °C (both in air and in N_2_ atmosphere), whereas the second stage (−26% decrease) occurred in the temperature range of 262–532 °C (air) (−6% decrease) and 262–315 °C (N_2_ atmosphere). The third stage of mass loss (−69% decrease) occurred in the temperature range of 532–662 °C (air, −22% decrease) and 315 to 900 °C (N_2_). The DTG curves differ only in the fourth event in air atmosphere (T_peak_ DTG 595 °C), which corresponds to the mass loss observed in the TG.

The DTA curve (Figure 2) showed the first endothermic event between 194–203 °C indicating melting (*T*_peak_ 197 °C, Heat −62 J·g^−1^), the second exothermic event between 209–218 °C indicating melting (*T*_peak_ 217 °C, Heat 518 J·g^−1^), the third exothermic event between 262–416 °C indicating melting (*T*_peak_ 288 °C, Heat 229 J·g^−1^), and the fourth one exothermic between 499–619 °C (*T*_peak_ 580 °C, Heat 7.44 kJ·g^−1^).

### 2.2. X-ray Fluorescence

X-ray fluorescence analysis shows the presence of some elements in the IVS320 sample, especially sodium, which is present at a concentration of 22% (Table 1). This relatively high amount of sodium might have been originated from the route of synthesis and isolation of this material, as well as from the reagents used in the synthesis process. In fact, anhydrous sodium sulfate was generated as solvent waste during IVS320 synthesis, which might explain the high sodium content. 

Other elements such as magnesium, aluminum, sulfur, and copper are also present, although in fairly low amounts (below 1‰). Just like sodium, these elements are chemical reagents used in the synthesis of IVS320. These results demonstrate that other than the original IVS320 molecule, there are additional chemical elements in the sample used in this study. 

X-ray fluorescence has been an important analytical tool for assessing the purity of drugs and excipients, being able to detect even small traces of contaminants, as demonstrated in this study.

### 2.3. X-ray Diffraction

**Figure 3:**
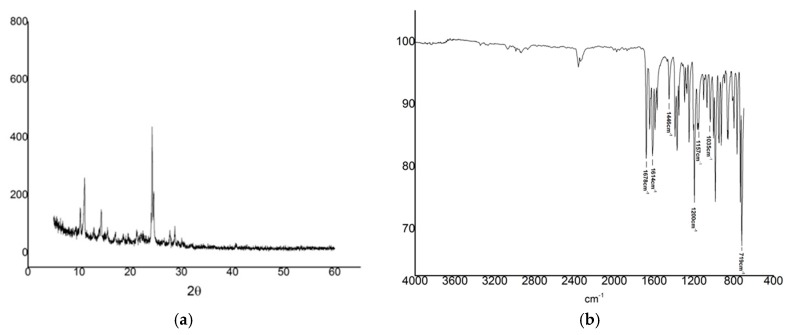
X-ray diffraction pattern (**a**) and Fourier-transform infrared (FT-IR) spectrum (**b**) of IVS320.

The X-ray diffractogram of IVS320 (Figure 3a) shows a series of high-intensity diffraction peaks at 10.30, 11.10, 14.34, 24.38 and 28.77° (2θ) in addition to several others secondary low-intensity peaks, indicating a crystalline structure. 

Additionally, its crystalline nature was confirmed with the result of the percentage of crystallinity (99.19%). Thus, the X-ray diffraction results indicate that the relatively high crystalline pattern of IVS320 might impair its solubility as highly crystalline compounds are usually poorly soluble in water [23].

### 2.4. Fourier Transform Infrared (FT-IR)

The FT-IR spectrum for IVS320 (Figure 3b) shows characteristic bands attributed to symmetrical carbonyl groups of naphthalene ring (stretching vibration at 1678 cm^−1^). Other bands were attributed to the C=C stretching vibration of cyclopentene (1614 cm^−1^) and the ones due to the benzene ring (1,2-disubstituted) at 1157 cm^−1^, 1035 cm^−1^ and 719 cm^−1^. In addition, a strong band is observed at 1200 cm^−1^ due to the asymmetrical stretching of C–O–C.

### 2.5. Scanning Electron Microscopy (SEM)

**Figure 4:**
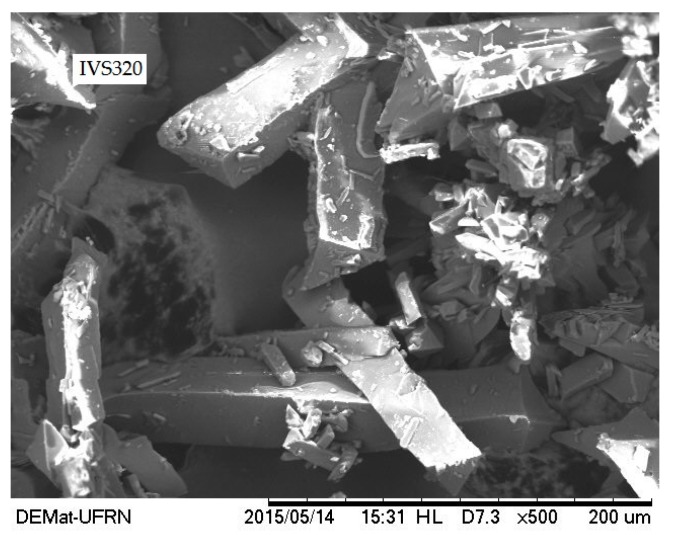
Scanning electron microscopy (SEM) micrograph of IVS320 at magnification of 500×.

SEM has been extensively used as a qualitative method to study the morphological aspects of solids. Figure 4 shows the micrograph of IVS320, where irregularly shaped crystals with various sizes and a predominance of a pyramidal form are observed. Such morphological findings are in accordance with those of naphthoquinone derivatives of 2-Hydroxy-1,4-naphthoquinone (lawsone), as previously reported [22]. The crystalline nature of IVS320 shown in the SEM micrograph corroborates the results of the X-ray diffraction analysis.

### 2.6. Hygroscopicity

Hygroscopicity can be defined as the tendency of a material to absorb moisture from the surrounding environment [24]. The compounds used for pharmaceutical applications are exposed to different humidity levels during the development stages such as synthesis, grinding, spray or freeze drying, wet milling, granulation, storage, and analysis [25].

Adsorption of water molecules may affect the stability, flow, dissolution, compressibility, and compatibility of powder mixtures used to manufacture pharmaceutical solid dosage forms [26,27]. Therefore, the interaction between the sample and water is of great interest in the preformulation studies of new drug candidates.

In this study, IVS320 was classified as a non-hygroscopic material according to the criteria depicted on Table 2 [28], as it shows a very low water adsorption capacity (H% < 0.01) regardless of the relative humidity condition (88.8% and 70.4%). In addition, the H% did not change from 3 to 72 h and therefore, it seems reasonable to infer that the H % reached its peak. These results indicate that the physical and physicochemical properties of IVS320 will not change even at high levels of humidity during storage and manufacturing.

### 2.7. Partition Coefficient/Lipophilicity

The permeation of any drug through biological membranes depends on its lipophilicity and therefore the drug’s absorption can be correlated with its partition coefficient [24].

The experimental lipophilicity (LogP) for IVS320 in 1-octanol/water system was 2.08 (standard deviation of 0.046, as shown in Table 3), which classifies it as a low permeability drug.

The computational LogP, which was calculated using the Marvin Sketch software, was 1.64. The proximity between the experimental and computational values seems to indicate the reliability of these methods.

Computational LogP for other naphthoquinone derivatives show lower values [29], which seems to indicate a decreased lipophilicity in comparison to IVS320.

### 2.8. Solubility

Solubility is a key factor that must be determined for any new chemical entity with potential as a drug candidate. This is because the drug’s dissolution rate and hence its bioavailability is a function of the solubility of the molecule [24].

In this study, qualitative analysis shows that IVS320 is insoluble in water and in most organic solvents tested. However, it is soluble in acetic acid, acetic anhydride, and acetone, where these last two are the most used solvents for IVS320 analytical and extraction purposes.

In order to predict the solubility of IVS320, a calibration curve was obtained using solutions at six different concentrations (10, 15, 20, 25, 30 and 35 µg/mL), where the equation y = 0.49x + 0.022, with R^2^ = 0.994 was then obtained. By using this equation, the quantitative solubility in water was calculated as 0.0121 μg/mL (standard deviation of 0.0013, as shown in Table 4), which classifies this drug as poorly soluble or insoluble in water, a result that confirms the qualitative solubility data.

Such poor aqueous solubility must be due to IVS320’s low polarity and high degree of crystallinity, as evidenced by the X-ray diffraction analysis (Figure 3a). In fact, the low water solubility of this drug is not an uncommon feature, as an estimated 40% of new chemical entities are lipophilic and therefore are poorly soluble in water [30].

## 3. Trypanocidal Activity

**Figure 5:**
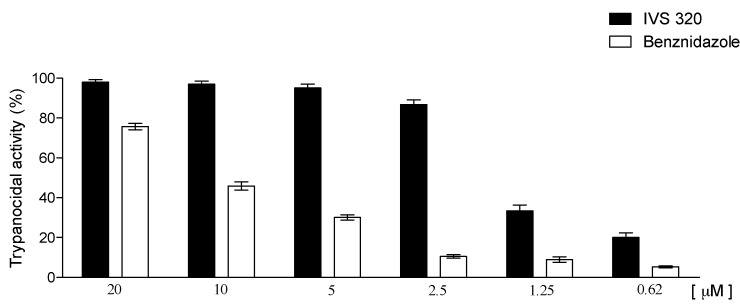
Effect of IVS320 upon the viability of trypomastigotes.

The trypanocidal activity of IVS320 was evaluated against tissue culture trypomastigotes forms of *T. cruzi* (Y strain) through the determination of IC_50_. IVS320 showed a potent anti*-T. cruzi* activity, being able to efficiently lyse the trypomastigotes forms. The IC_50_ of IVS320 was 1.49 ± 0.1 µM against 11.4 ± 1.4 µM of the reference drug benznidazole (Figure 5).

IVS320 was even more active than the analogs originated from the insertion of 1,2,3-triazole in the 1,4-naphthoquinone structure, where they showed IC_50_ that ranged from 10 to 17 µM [31]. In addition, IVS320 was the most effective among the 2-aminonaftoquinone derivatives in which, despite the increased time of exposure to the parasite, showed IC_50_ values around 7.5 µM [32].

**Figure 6:**
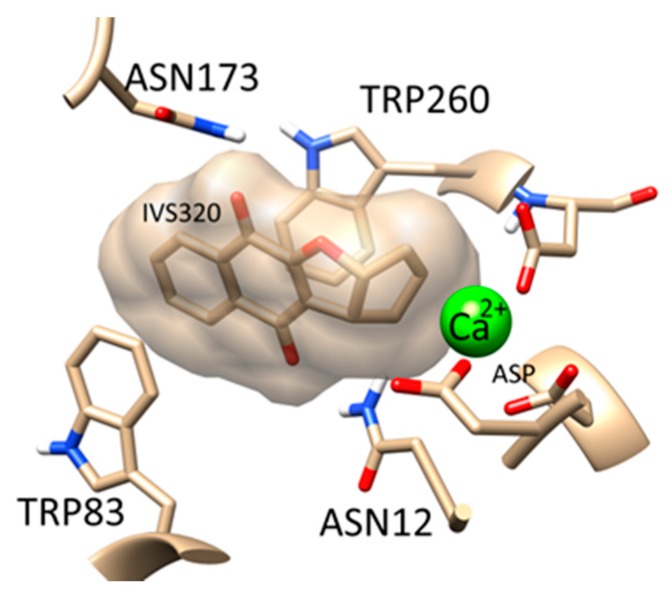
Possible binding mode of IVS320 in the Trypanosomatidae nucleoside hydrolase target.

In order to identify potential biological activities of IVS320, reverse virtual screening was performed in more than 9000 targets. The results showed that IVS320 was strongly bound to two targets: Nucleoside hydrolase (PDB ID: 1HP0) [33,34] and pteridine reductase (PDB ID: 3JQB) [35,36], where both are unique to Trypanosomatidae. The high affinity of IVS320 for these proteins might explain the efficient trypanocidal activity reported in this study. Figure 6 shows a possible binding mode for IVS320 with nucleoside hydrolase from *T. cruzi*.

## 4. Materials and Methods

### 4.1. Material 

IVS320 (3*a*,10*b-*dihydro-1*H*-cyclopenta[*b*]naphtho[2,3-*d*]furan-5,10-dione) was synthesized by the research group at Universidade Federal Fluminense following their previoulsy reported method [19]. The experiments were conducted using ultrapure water (MILLI Q). Other reagents and chemicals were of analytical grade.

### 4.2. Differential Scanning Calorimetry (DSC)

DSC curves were obtained in a Shimadzu DSC-60 cell, using closed aluminum pans with around 2 mg of IVS320, under dynamic atmosphere of N_2_ (flow rate of 50 mL·min^−1^) and heating rate of 10 °C·min^−1^ in the temperature range of 25–450 °C. 

Highly pure Zn and In were used to calibrate the DSC equipment, where the experiments were performed at 200 and 500 °C, respectively. Through their melting points (156.65 and 419.50 °C for In and Zn, respectively) the areas under the peaks were determined. Once the correction of the calibration temperature was performed, the heat calibration was corrected in which the enthalpy values for In and Zn were 28.5 and 100.5 J·g^−1^, respectively. Further, new experiments were performed to assure that the melting temperature varied in the range of ±0.5 °C and that the values of melting enthalpy (Δ*H*) varied in the range of ±1.0 J·g^−1^. Once these parameters were reached, the calibration was accomplished.

### 4.3. Termogravimetry & Differential Thermal Analysis (TG and DTA)

TG and DTA curves for IVS320 were obtained on a SHIMADZU thermobalance, model TGA 60 (simultaneous TG/DTA), using an alumina pan (samples weighting 8 ± 0.1 mg) at a heating rate of 10 °C·min^−1^ in the 25–900 °C temperature range, under dynamic atmosphere of Air and N_2_ at 50 mL·min^−1^.

The TGA 60 equipment was calibrated using In, which was heated up to 200 °C followed by correction of the calibration temperature. Next, another experiment was run with the purpose of checking whether the melting temperature varied within ±0.5 °C.

In order to identify the thermal events presented as well as the temperatures (*T*_onset_, and *T*_peak_) and energies (J·g^−1^) involved in these events, thermal curves were analyzed with the aid of the SHIMADZU software TASYS. 

### 4.4. X-ray Fluorescence

For the X-ray fluorescence analysis, a X-ray Fluorescence by Dispersion of Energy (XRFDE) spectrometer, model EDX-700 (Shimadzu^®^), was used. X-ray data were obtained using a Rhodium tube with voltage from 0 to 40 KeV, collected after 250 s. All chemical elements were identified by their Kα or Lα energies and the quantification was performed based on the relative intensities of cps/uA.

The quantification of the elements found in IVS320 was performed using external high-purity standards at six predetermined concentrations, which were subjected to the same analysis protocol.

### 4.5. X-ray Diffraction

X-ray diffraction analysis were performed using a XRD 6000 (Shimadzu^®^) with CuKα radiation generated at 40 kV and 30 mA, 2.0 speed 0/min, 0.020 pitch, with scanning from 10 to 600. Crystallinity of the material was calculated using the following equation:

(1) $$\mathrm{crystallinity}=\frac{1}{1+K\times \frac{I_{a}}{I_{cr}}}$$

where the integral intensities of the crystalline and amorphous parts are I_cr_ and I_a_, respectively, and the ratio of X-ray intensity of the amorphous part, which scatters from a fixed amount of material, to that of its crystalline part is K.

### 4.6. Fourrier Transform Infrared (FT-IR)

The FT-IR spectrum (4000–400 cm^−1^) was obtained on a Prestige-21 FT-IR spectrophotometer (Shimadzu^®^) equipped with a selenium crystal. The number of scans was 120 and the resolution was 4 cm^−1^.

### 4.7. Scanning Electron Microscopy (SEM)

The morphology of IVS320 powder was obtained by SEM. Sample was fixed on stub with double carbon tape and imaged on a Tabletop Microscope TM300 (Hitachi^®^) at a magnification of 500×.

### 4.8. Hygroscopicity

Hygroscopicity was determined according to the method described by Callahan and co-workers [28] with some modifications. Briefly, 50 mg of IVS320 was subjected to different relative humidity conditions (88.8% and 70.4%), which were obtained by using sulfuric acid solution at 4.92 and 8.68 N, respectively, for 3, 24, 48 and 72 h. 

The hygroscopicity of IVS320 was determined as percentage of water absorbed (H %), which was calculated using the following equation:

(2) H % =[(Wh – Wd)/Wd] × 100

where Wh and Wd are the weight of the sample after (humid) and before (dried) submitting IVS320 to a fixed relative humidity environment.

### 4.9. Partition Coefficient/Lipophilicity

Partition coefficient of IVS320 was determined according to the slow-stirring method adapted for poorly soluble drugs [37]. In this experiment, 1-octanol and distilled water were used as the organic and aqueous phases, respectively, where the two phases were subjected to saturation for two days prior to the experiment.

IVS320 was added to the 1-octanol/water system and subjected to slow stirring at room temperature for 5 days. The turbulence between the two phases was controlled to prevent emulsification. IVS320 was quantified at the aqueous and octanolic phases by spectrophotometry in the UV region (268 nm). The partition coefficient (P) was calculated as the ratio between the concentration of IVS320 in the 1-octanol and water phases, P = [1-octanol]/[water]. Lipophilicity, denoted as log P, was measured as the base 10 logarithm of P. The experiment was performed in triplicate.

### 4.10. Solubility Studies

The qualitative solubility of IVS320 was determined using solvents with different polarities: water, acetonitrile, methanol, ethanol, dichloromethane, ethyl ether, oleic acid, dipropylene, ortho-phosphoric acid, hexane, chloroform, petroleum ether, glycerin, folic acid, acetic anhydride, acetic acid, and acetone. A fixed amount of IVS320 (1 mg) was added to different volumes of each solvent (1, 10, 100 and 1000 mL) and subjected to vigorous mechanical stirring at 25 °C (±2 °C) for 12 h. The obtained dispersion was filtered (0.45 μm) and the amount of IVS320 dissolved was determined spectrophotometrically at 268 nm. This experiment was performed in triplicate.

### 4.11. Parasites

Tissue culture trypomastigotes (Y strain) were obtained from the supernatants of 5 to 6-days-old infected LLC-MK2 cells maintained in RPMI-1640 medium supplemented with 10% FBS (FBS; Cultilab, Campinas, Brazil) and 50 µg/mL gentamycin (Novafarma, Anápolis, Brazil) at 37 °C in a 5% humidified CO_2_ atmosphere.

### 4.12. Anti-Parasitic Activity 

Tissue culture trypomastigotes obtained from the supernatants of previously infected LLC-MK2 cells were dispensed into 96-well plates (4 × 10^5^ cell/well) in RPMI medium supplemented with 10% FBS and 50 µg/mL of gentamycin in the absence or in the presence of different concentrations (20–0.15 µM) of IVS320, in triplicate. Viable parasites were counted in a Neubauer chamber at 24 h after incubation. The percentage of inhibition was calculated in relation to untreated cultures. To determine the inhibitory concentration of 50% (IC_50_) for trypomastigote forms of *T. cruzi*, a nonlinear regression on Prism 5.02 GraphPad software was used. Experiments were performed in triplicate and benznidazole (LAFEPE, Recife, Brazil) was used as trypanocidal reference drug.

### 4.13. Inverse Virtual Screening

Around 9000 targets with known ligands were retrieved from the Protein DataBank [38], preprocessed and used to dock IVS320. AutoDock Vina [39] was used for this process. The most favorable binding energies were analyzed and Trypanosomatidae targets were selected and visualized using UCSF Chimera [40].

## 5. Conclusions 

This study provided important information regarding the physicochemical properties of the new chemical entity IVS320, which was found to be insoluble in water and in most organic solvents. Its high degree of crystallinity (99.19%) may have contributed to such poor solubility. Although the antitumor and antifungal activities of IVS320 have been documented, this study demonstrated that this drug has a potent trypanocidal activity against tissue culture trypomastigotes and therefore has potential as a new anti-trypanosoma drug.

This work opens perspectives for future studies attempting to improve IVS320’s physical and chemical characteristics, especially solubility and permeability, through inclusion complexes and solid dispersions. Moreover, the in vivo activity of IVS320 needs to be investigated in order to better elucidate its mechanisms of action and further treatment of other infectious diseases.

## Figures and Tables

**Table 1:** X-ray fluorescence analysis of IVS320.

Chemical Element	Concentration ‰ (*w*/*w*)
**Na**	22.13
**Mg**	0.579
**Al**	0.615
**S**	0.03
**Cu**	0.01

**Table 2:** Hygroscopicity classification [28].

Classification	Criteria
Non-hygroscopic	No moisture increase at humidity levels below 90%. Less than 20% (*w*/*w*) increase in moisture content at humidity levels above 90% after 1 week of storage.
Slightly hygroscopic	No moisture increase at humidity levels below 80%. Less than 40% (*w*/*w*) increase in moisture content at humidity levels above 80% after 1 week of storage.
Moderately hygroscopic	Moisture content does not increase >5% (*w*/*w*) at humidity levels below 60%. Less than 50% (*w*/*w*) increase in moisture content at humidity levels above 80% after 1 week of storage.
Very hygroscopic	Moisture content increases at humidity levels as low as 40–50%. Greater than 20% (*w*/*w*) increase in moisture content at humidity levels above 90% after week of storage.

**Table 3:** Results of LogP from the triplicate experiments.

Data	LogP *
**1**	2.04
**2**	2.13
**3**	2.07

* Standard deviation was calculated as 0.046.

**Table 4:** Results of solubility of IVS320 from the triplicate experiments.

Data	Solubility (μg/mL) *
**1**	0.0108
**2**	0.0134
**3**	0.0122

* Standard deviation was calculated as 0.0013.
